# Prognostic significance and temporal patterns of glycemic variability in critically ill non-diabetic patients with ischemic stroke: a retrospective multicenter cohort study

**DOI:** 10.3389/fneur.2026.1825235

**Published:** 2026-06-12

**Authors:** Jiani Sang, Weizhao Li, Zhaoyang Feng, Jiale Kang, Danyang Niu, Junjie Liu

**Affiliations:** 1 School of Clinical Medicine, North China University of Science and Technology, Tangshan, Hebei, China; 2 School of Stomatology, North China University of Science and Technology, Tangshan, Hebei, China; 3 Affiliated Hospital of North China University of Science and Technology, Tangshan, Hebei, China

**Keywords:** all-cause mortality, glycemic variability, intensive care unit, ischemic stroke, machine learning

## Abstract

**Background:**

Critically ill patients with non-diabetic ischemic stroke (ND-IS) often exhibit significant blood glucose fluctuations, but traditional glycemic indicators fail to capture such dynamic changes. Evidence regarding the association of glycemic variability (GV) with outcomes in this patient population remains limited. In critically ill patients with ND-IS, this study sought to investigate the association of GV with all-cause mortality (ACM) at multiple follow-up points.

**Methods:**

This multicenter cohort study analyzed 2,971 ND-IS patients from MIMIC-IV database (original cohort) and Affiliated Hospital of North China University of Science and Technology (validation cohort) between December 2020 and November 2024. GV was calculated as the coefficient of variation (CV), and participants were divided into quartiles (Q1-Q4). Survival analysis (Kaplan–Meier) and multivariable Cox proportional hazards models were used to evaluate the independent association of GV with 28-, 90-, 180-, and 365-day ACM. Nonlinearity was evaluated with restricted cubic splines, and predictive performance was assessed via ROC curves, decision-curve analysis, net reclassification improvement (NRI), and integrated discrimination improvement (IDI).

**Results:**

In the original cohort, the median age was 70 years (IQR 59–80), and 51.9% of patients were male. ACM increased progressively across GV quartiles (Q4 vs. Q1: 28-day 25.1% vs. 11.2%; 365-day 32.0% vs. 12.8%; both *p* < 0.001). In fully adjusted models, high GV remained independently associated with an increased risk of mortality (28-day HR = 1.31, 95% CI 1.03–1.65; 365-day HR = 1.49, 1.24–1.79; both *p* < 0.05). Spline analysis suggested a nonlinear association: mortality risk increased steeply beyond GV of approximately 16–18% and appeared to plateau around 36–38%. Adding GV to conventional severity models improved long-term prognostic accuracy (NRI and IDI both *p* < 0.05).

**Conclusion:**

Elevated GV is independently associated with increased short- and long-term ACM in critically ill patients with ND-IS. GV suggested a time-dependent association with both survival curves and dynamic physiological alterations in ND-IS patients. As a dynamic metabolic marker, GV highlights the potential of this dynamic metric to inform future strategies for risk assessment in critical care.

## 1 Introduction

Ischemic stroke (IS), which constitutes approximately 80% of all stroke events, represents a major global health challenge and a primary contributor to mortality and morbidity (1). Among critically ill patients with non-diabetic ischemic stroke (ND-IS), acute brain injury–induced neuroendocrine disturbances often result in marked stress-related fluctuations in blood glucose, even without pre-existing diabetes and insulin resistance (2).

Conventional blood glucose metrics offer an incomplete picture of metabolic control in critically ill patients. They provide a limited, static assessment that fails to capture the dynamic nature of glycemic fluctuations. For instance, while HbA1c reflects a 2–3 months average glucose level, it cannot detect acute glycemic swings during an ICU stay (3). Similarly, the hemoglobin glycation index (HGI) measures the deviation between observed and HbA1c-predicted glucose levels but lacks the sensitivity to track rapid changes under stress responses (4). The stress hyperglycemia ratio (SHR) is another limited tool, which based on the admission-to-baseline glucose ratio. It assesses the severity of hyperglycemia at admission but ignores the frequency and amplitude of glucose variations over time (5). In contrast, glycemic variability (GV) provides a more comprehensive and clinically relevant measure. By quantifying both the magnitude and frequency of glucose oscillations, GV captures the dynamic glycemic fluctuations that is characteristic of critical illness (6, 7).

Recent research increasingly reports a potential relationship between GV and poorer clinical outcomes among patients with cardiovascular events, infections, renal failure, and critical illness (8, 9). However, most previous studies have combined diabetic and ND-IS patients into a single analysis group (10). This combined approach overlooks the distinct metabolic mechanisms underlying stress-induced glucose fluctuations in non-diabetic cases (e.g., the absence of pre-existing insulin resistance). Although some subgroup analyses suggest GV may have a stronger prognostic effect in non-diabetic individuals, this hypothesis has not been systematically explored (11–13). Consequently, applying these generalized findings specifically to critically ill patients with ND-IS remains problematic. Many studies assess glycemic variability using single indicators (e.g., standard deviation) (14) rather than more comprehensive measures (e.g., coefficient of variation or mean amplitude of glycemic excursions). Those single fail to provide a comprehensive characterization of glycemic instability. Furthermore, most research focuses on short-term outcomes like in-hospital or 28-day mortality, with few having examined long-term endpoints such as 365-day all-cause mortality (ACM) (15). This gap leaves the sustained impact of GV on patient prognosis unclear. Key pathophysiological mechanisms like oxidative stress and inflammatory responses have not yet been systematically validated in patients with ND-IS (16).

The aim of this multicenter cohort study was to assess the link between GV and ACM across multiple time points in critically ill patients with ND-IS. We placed specific emphasis on identifying potential nonlinear relationships, threshold effects, and time-dependent patterns. A further objective was to assess the independent prognostic significance of GV compared to conventional disease severity scores. The findings are expected to provide evidence-based guidance for individualized glycemic management in critically ill patients with ND-IS.

## 2 Materials and methods

### 2.1 Data source

Data for the original cohort were obtained from the Medical Information Mart for Intensive Care IV (MIMIC-IV, version 3.1), a clinical database curated and maintained by the Laboratory for Computational Physiology at the Massachusetts Institute of Technology (MIT). This database represents a large, publicly accessible clinical repository encompassing data from intensive care unit (ICU) patients (17). The de-identified health records within the MIMIC-IV database comprise over 90,000 ICU admissions recorded between 2008 and 2022. The Institutional Review Boards (IRBs) of MIT and Beth Israel Deaconess Medical Center granted joint approval for the use of this publicly available database. Accordingly, the requirement for informed consent was waived for this analysis (IRB approval No. 2001-P-001699/14; ID No. 0403000206). One researcher (JL) obtained certified access to the database (certification number: 52698592) and has extensive experience in data extraction and management using the MIMIC database (18, 19).

Data for the validation cohort were obtained from Affiliated Hospital of North China University of Science and Technology. We consecutively enrolled eligible patients admitted to ICU between November 2022 and November 2024. This study was reviewed and approved by the hospital’s Clinical Research Ethics Committee (IRB approval No. 20240930057). These analysis of human data was conducted in accordance with institutional and national ethical standards and adhered to the principles of the Declaration of Helsinki (1964) and its subsequent amendments.

### 2.2 Study population

In this study, ND-IS was defined as an acute ischemic stroke occurring in adults with no history of diabetes mellitus. The diagnosis of ischemic stroke was established in accordance with the 2019 American Heart Association/American Stroke Association (AHA/ASA) Guidelines (reference for the validation cohort) and identified using International Classification of Diseases (ICD) codes (for the derivation cohort) (20). Non-diabetic status was defined by the absence of a documented diabetes history and an admission glycated hemoglobin (HbA1c) level <6.5%, consistent with the American Diabetes Association (ADA) criteria (21).

For the original cohort, the inclusion criteria were: adult patients diagnosed with ischemic stroke at ICU admission, identified via ICD-9 or ICD-10 codes. The exclusion criteria were: (1) a diagnosis of diabetes mellitus or related complications (defined by ICD-9/10 codes); (2) age under 18 years; (3) fewer than three blood glucose measurements during the ICU stay; (4) an ICU stay of less than 24 h; (5) multiple ICU admissions, in which case only the first admission was analyzed.

**Figure 1:**
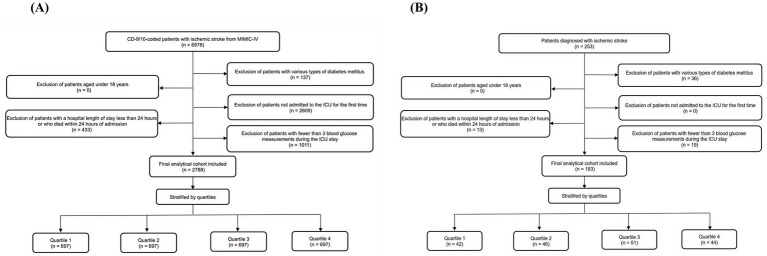
Flowchart of patient selection and study workflow. **(A)** Patient screening for the original cohort (MIMIC-IV ICD-coded ischemic stroke patients stratified into four quartiles); **(B)** Patient screening for the validation cohort (AHA/ASA guideline-confirmed acute ischemic stroke patients with quartile stratification).

For the validation cohort, the inclusion criteria were: adult patients with a diagnosis of acute ischemic stroke confirmed by head CT or MRI per the 2019 AHA/ASA Guidelines (20). The exclusion criteria were: (1) a documented history of diabetes mellitus or an admission HbA1c level ≥ 6.5% (per ADA criteria (21)); (2) age under 18 years; (3) fewer than three blood glucose measurements during the ICU stay; (4) an ICU stay of less than 24 h; (5) multiple ICU admissions, in which case only the first admission was analyzed. For the original (MIMIC-IV) cohort, non-diabetic status was defined by the absence of documented diabetes mellitus (via ICD-9/10 codes). Given the high rate of missing HbA1c data in this database, it was not utilized to define the cohort, nor was it included in the analytical models. A flow diagram of patient selection is presented in Figure 1, and the corresponding ICD-9/10 codes are provided in Supplementary Table S1.

### 2.3 Definition of GV

In this study, glycemic variability (GV) was defined as the fluctuation in blood glucose levels during the intensive care unit (ICU) stay. GV was quantified using the coefficient of variation (CV), a standard metric for assessing dispersion, calculated as: $\mathrm{GV}=(\frac{\mathrm{SD}}{\mathrm{MBG}})\times 100\%$ (SD, standard deviation; MBG, mean blood glucose) (22). To ensure a stable estimate, we included only patients with at least three glucose measurements during their ICU admission. The timing of these measurements was not protocol-specified but followed clinical routine, thereby encompassing the entire ICU stay and reflecting real-world monitoring patterns. Blood glucose values were obtained from routine clinical measurements during the ICU stay, encompassing arterial, venous, and capillary sources as per standard care.

The coefficient of CV was selected as the primary metric for GV because it expresses glucose variability relative to the mean level. This relative measure (SD/MBG × 100%) allows for more comparable assessment of glycemic fluctuations across patients with differing average glucose concentrations, which is particularly relevant in a critically ill population.

### 2.4 Patient characteristics

The collected covariates were as follows (Supplementary Table S2): (1) demographic characteristics, such as age, sex, and ethnicity; (2) comorbid conditions, such as heart failure (HF), malignancy (CA) and chronic kidney disease (CKD); (3) laboratory parameters, such as international normalized ratio (INR), electrolytes (Na, K, Ca, Mg, Cl) and bicarbonate levels; (4) vital signs, such as heart rate (HR) and arterial oxygen saturation (SpO2); (5) medications, such as antiplatelet agents, insulin, and vasopressin; (6) disease severity scores, such as Sequential Organ Failure Assessment (SOFA) and Shock index; and (7) therapeutic interventions, such as continuous renal replacement therapy (CRRT), and mechanical ventilation (MV). All laboratory parameters and disease severity scores were obtained from the first recorded measurements within the first 24 h after intensive care unit admission.

### 2.5 Clinical outcomes

We defined ACM at 28, 90, 180, and 365 days after ICU admission as the primary study outcomes. The ICU and hospital length of stay were analyzed descriptively only.

### 2.6 Statistical analysis

Variables were retained for analysis only if the proportion of missing values was 20% or less, and the patterns of missingness are presented in Supplementary Figure S1. To handle incomplete variables with under 20% missing values, multiple imputation was employed to complete the dataset (*m* = 5 imputations) (23). Predictive mean matching (PMM) was employed for continuous variables, logistic regression models for binary variables, and a classification and regression tree (CART) algorithm for categorical variables. Multiple imputation effectively preserves data variability and yields more accurate estimates of standard errors. Potential outliers were initially identified using boxplots. All identified potential outliers were manually verified, and values clearly outside the physiological range were excluded; remaining outliers were imputed using the median.

In this study, Normality for all continuous data was assessed via the Shapiro–Wilk test. As most clinical variables exhibited a non-normal distribution, continuous variables were expressed as the median (M) and interquartile range (IQR) in subsequent analyses. According to the distribution of GV in this cohort, the 25th, 50th, and 75th percentiles (10.8, 16.27, and 24.07%, respectively) were used as cut-off values to categorize patients into four quartiles: Q1 (GV ≤ 10.8%), Q2 (10.8% < GV ≤ 16.27%), Q3 (16.27% < GV ≤ 24.07%), and Q4 (GV > 24.07%). If the assumptions of normality and homogeneity of variance were satisfied, independent-samples t-tests (for two groups) or ANOVA (for ≥3 groups) were employed to compare continuous variables across groups. Otherwise, the non-parametric Mann–Whitney U test (two groups) or Kruskal-Wallis H test (multiple groups) was employed. Categorical variables were summarized as frequencies and percentages (%), and Pearson’s chi-square test was employed to assess group differences. Initial assessing for multicollinearity among the variables was employed with Spearman’s rank correlation. Variables with correlation coefficients (*ρ*) greater than 0.5 were considered moderately correlated and were excluded from subsequent modeling. We used the variance inflation factor (VIF) to evaluate the remaining variables for potential collinearity (24). We then applied least absolute shrinkage and selection operator (LASSO) regression and the Boruta algorithm to identify predictive variables. This process identified key factors potentially associated with ACM in ND-IS patients and confirmed GV’s independent predictive value.

We constructed Kaplan–Meier (K–M) survival curves stratified by GV quartiles to assess survival differences across groups. Next, we used the surv_cutpoint function from the R package survminer to determine the optimal cut-off points for risk stratification across the four mortality endpoints. Group differences were compared using the log-rank test.

We built stepwise Cox proportional hazards models to assess hazard ratios (HRs) for GV, using the Q1 quartile as the reference. Model 1 (crude model) contained only the GV quartile. Model 2 added adjustments for demographic factors: age, sex, and ethnicity. Model 3 (fully adjusted model): further adjusted for vital signs (HR, RR and DBP); severity scores (SOFA and GCS), comorbidities (Myocardial infarction, HF, COPD, CKD, and malignancy), treatments (CRRT and MV); and medication (vasopressors, *β*-blockers, statins, and antiplatelet agents). The proportionality assumption of the Cox model was tested with Schoenfeld residuals. To mitigate overfitting in the validation cohort, the fully adjusted Model 3 was not re-estimated. We directly applied the covariate set and coefficient estimates from the original cohort to this cohort, assessing the transportability of the association under a fixed model.

To explore the potential nonlinear association of GV with ACM, as well as to examine possible threshold and saturation effects, we used a four-knot restricted cubic spline (RCS) model. The statistical significance of nonlinearity was evaluated using the Wald test. We also conducted subgroup analyses to examine the association of GV with prespecified variables in relation to mortality risk by prespecified variables, including age (>65 or ≤65 years), sex, race, CAD, CKD, CA, MI, COPD, heart failure, hypertension, and AF.

To compare the predictive performance of GV with traditional prognostic scores, including SOFA, APS III, SIRS, OASIS, APACHE II, Pathos score, and SAPS II for ACM, we constructed a reference model that contained all covariates from Model 3 except for any components of the traditional scores being compared. The predictive performance of this reference model augmented with either GV or a single traditional score was then compared using the area under the receiver operating characteristic curve (AUC) with 95% confidence intervals and determined clinical net benefit using decision curve analysis (DCA). A comparison of AUCs between models was made using the DeLong test. The net reclassification improvement (NRI) and integrated discrimination improvement (IDI) were calculated to assess the incremental predictive value (IPV) of adding GV to the reference model. Model optimism was assessed via bootstrapping (500 replicates) for internal validation.

Additionally, we conducted several sensitivity analyses to ensure the robustness of our findings. First, we used boxplots to identify outliers in the GV data, defining them as values exceeding 1.5 times the interquartile range (IQR). Outliers represented approximately 4% of all data points and clustered mainly in the upper distribution. To reduce their impact, a Winsorization technique was applied to cap the extreme GV values at the 96th percentile. Then the data were recategorized into new quartiles. To address potential reverse causality, we excluded patients who died within the initial 72 h of ICU admission and regrouped the cohort based on the updated GV quartiles. We also conducted stratified analyses by ICU length of stay (≤3 days vs. >3 days) to assess whether GV’s predictive stability varied by hospitalization time. Additionally, we adjusted the full Model 3 for the total number of blood glucose measurements, mean sampling interval, and overall sampling duration to account for potential bias due to variations in sampling frequency. All statistical analyses were performed with R software (version 4.2.2) and DecisionLinnc (version 1.0), and statistical significance was defined by a two-sided *p*-value < 0.05.

## 3 Results

### 3.1 Baseline characteristics of cohort

**Figure 2:**
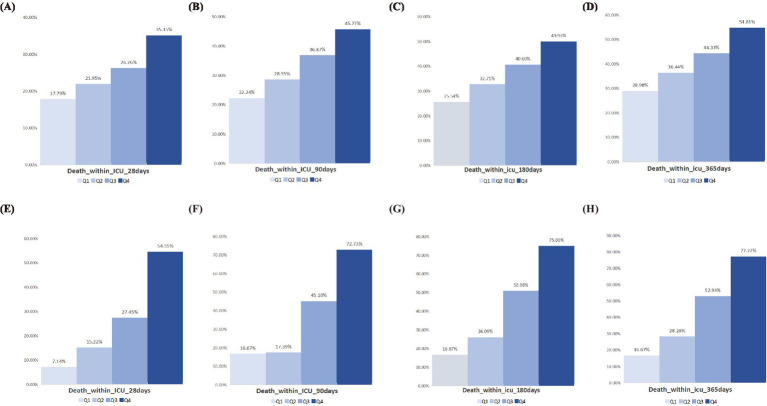
Comparison of ACM rates across GV quartiles. **(A–D)** Original cohort: ACM proportion at 28, 90, 180, and 365 days after ICU admission. **(E–H)** Validation cohort: Corresponding ACM proportion at the same time points. Patients are stratified by GV quartiles (Q1–Q4).

The study consisted two cohorts: an original cohort of 2,788 patients from the MIMIC-IV database and a validation cohort of 183 patients from Affiliated Hospital of North China University of Science and Technology. A total of 2,971 patients with ND-IS were included in the analysis (Table 1 and Supplementary Table S3). Based on the quartiles of GV, the study cohort was divided into four groups in the original cohort: Q1 (GV ≤ 10.8%, *n* = 697), Q2 (10.8% < GV ≤ 16.27%, *n* = 697), Q3 (16.27% < GV ≤ 24.07%, *n* = 697), and Q4 (GV > 24.07%, *n* = 697). The overall median GV was 16.27% (IQR, 10.80–24.07), and its distribution is presented in Supplementary Figure S2. The median age of the cohort was 70 years (IQR, 59.00–80.00), with males comprising 51.9% and females 48.1%. Sex distribution differed significantly among GV groups (*p* = 0.018), with a greater percentage of males in the lower GV quartiles (Q1 and Q2: 54.1 and 54.7%, respectively) and a higher proportion of females in the highest quartile (Q4: 52.9%). With increasing GV levels, SBP and DBP gradually decreased, whereas RR and HR exhibited an upward trend (all *p* < 0.001), indicating that patients in the high GV group might experience more pronounced circulatory stress responses. Regarding comorbidities, patients in the high GV group had higher prevalences of metabolic and cardiovascular conditions, including anemia, renal insufficiency, Coronary artery disease, Acute kidney injury, Liver cirrhosis, CKD, HF, Myocardial infarction, and COPD (all *p* < 0.001). Hypertension (*p* = 0.002), atrial fibrillation (*p* = 0.020), and COVID-19 (p = 0.020) were also significantly more common among those with high GV. In terms of laboratory parameters and disease severity, higher GV levels were significantly associated with elevated scores on the SOFA, APS III, SAPS II, OASIS, CCI, and APACHE II scales, as well as higher shock indices (all *p* < 0.001). Patients in the high GV group demonstrated more pronounced inflammatory, coagulation, and metabolic abnormalities, characterized by lower hematocrit, hemoglobin, and serum calcium levels. Conversely, white blood cell count, RDW, INR, PT, serum Potassium (*p* = 0.002), chloride (*p* = 0.046), glucose, creatinine, BUN, and anion gap levels were all significantly higher (all *p* < 0.001). For interventions, patients in the high GV group received significantly higher proportions of CRRT, MV, antibiotics, glucocorticoids, *β*-blockers, insulin, antihypertensive agents, and vasopressin (all *p* < 0.001). As shown in Figure 2 and Supplementary Figure S3, higher GV levels were associated with progressively increased 28-day, 90-day, and 365-day ACM rates (all *p* < 0.001), along with significantly prolonged ICU and hospital stays (*p* < 0.001).

**Table 1:** Baseline characteristics of ND-IS patients stratified by GV quartiles in original cohort.

Categories	Overall	Q1	Q2	Q3	Q4	*p*
	*N* = 2,788	*N* = 697	*N* = 697	*N* = 697	*N* = 697	
Age	70.00 (59.00, 80.00)	69.00 (58.00, 80.00)	71.00 (60.00, 82.00)	71.00 (58.00, 81.00)	70.00 (60.00, 80.00)	0.342
Sex						0.018
Female	1,341.00 (48.10%)	320.00 (45.91%)	316.00 (45.34%)	336.00 (48.21%)	369.00 (52.94%)	
Male	1,447.00 (51.90%)	377.00 (54.09%)	381.00 (54.66%)	361.00 (51.79%)	328.00 (47.06%)	
Race						0.329
Other	1,192.00 (42.75%)	282.00 (40.46%)	293.00 (42.04%)	302.00 (43.33%)	315.00 (45.19%)	
white	1,596.00 (57.25%)	415.00 (59.54%)	404.00 (57.96%)	395.00 (56.67%)	382.00 (54.81%)	
Weight, kg	77.00 (65.00, 91.90)	77.60 (66.50, 93.35)	78.00 (65.20, 92.50)	77.00 (64.65, 91.00)	74.80 (63.40, 90.70)	0.07
Vital sign						
SBP, mmHg	128.14 (114.40, 142.68)	133.83 (120.00, 147.74)	129.92 (115.83, 142.07)	125.63 (112.62, 141.00)	123.21 (110.50, 138.64)	<0.001
RR, beats/min	18.88 (16.88, 21.50)	18.32 (16.63, 20.38)	18.96 (16.89, 21.43)	19.15 (17.10, 21.73)	19.12 (17.10, 22.59)	
HR, beats/min	81.46 (71.64, 92.88)	78.18 (69.10, 87.96)	81.46 (70.69, 93.45)	82.20 (72.48, 93.04)	84.19 (74.26, 95.48)	<0.001
DBP, mmHg	85.67 (75.98, 96.06)	90.00 (80.14, 99.96)	86.50 (76.50, 96.77)	83.09 (74.81, 94.28)	82.75 (73.65, 92.00)	<0.001
SpO₂, %	97.29 (95.96, 98.56)	96.96 (95.77, 98.28)	97.29 (95.96, 98.52)	97.37 (95.96, 98.72)	97.41 (96.19, 98.71)	<0.001
Temperature, °F	98.45 (98.08, 98.92)	98.43 (98.11, 98.82)	98.48 (98.10, 98.96)	98.41 (98.07, 98.93)	98.47 (98.05, 99.02)	0.691
Severity score						
SOFA	4.00 (2.00, 6.00)	2.00 (1.00, 4.00)	4.00 (2.00, 6.00)	4.00 (2.00, 7.00)	5.00 (3.00, 8.00)	<0.001
APS III	41.00 (30.00, 55.00)	33.00 (24.00, 43.00)	39.00 (30.00, 52.00)	44.00 (32.00, 57.00)	49.00 (37.00, 65.00)	<0.001
SIRS	3.00 (2.00, 3.00)	2.00 (2.00, 3.00)	3.00 (2.00, 3.00)	3.00 (2.00, 3.00)	3.00 (2.00, 3.00)	<0.001
SAPS II	35.00 (27.00, 44.00)	31.00 (23.00, 38.00)	35.00 (27.00, 43.00)	37.00 (29.00, 47.00)	40.00 (31.00, 51.00)	<0.001
OASIS	32.00 (27.00, 38.50)	29.00 (24.00, 35.00)	32.00 (27.00, 38.00)	33.00 (28.00, 39.00)	35.00 (29.00, 41.00)	<0.001
GCS	14.00 (11.00, 15.00)	14.00 (11.00, 15.00)	14.00 (11.00, 15.00)	14.00 (11.00, 15.00)	14.00 (11.00, 15.00)	0.054
CCI	7.00 (5.00, 9.00)	6.00 (4.00, 8.00)	6.00 (4.00, 8.00)	7.00 (5.00, 9.00)	7.00 (5.00, 9.00)	<0.001
Pathos score	2.00 (1.00, 3.00)	2.00 (1.00, 2.00)	2.00 (1.00, 3.00)	2.00 (2.00, 3.00)	3.00 (2.00, 3.00)	<0.001
APACHE II	16.00 (12.00, 22.00)	13.00 (10.00, 18.00)	16.00 (12.00, 21.00)	17.00 (13.00, 22.00)	19.00 (14.00, 25.00)	<0.001
Shock index	1.00 (0.77, 1.29)	0.83 (0.66, 1.07)	0.98 (0.77, 1.25)	1.04 (0.82, 1.38)	1.14 (0.92, 1.44)	<0.001
Comorbidity, *n* (%)						
CHF						0.03
No	2,546.00 (91.32%)	654.00 (93.83%)	637.00 (91.39%)	631.00 (90.53%)	624.00 (89.53%)	
Yes	242.00 (8.68%)	43.00 (6.17%)	60.00 (8.61%)	66.00 (9.47%)	73.00 (10.47%)	
Anemia						<0.001
No	1,799.00 (64.53%)	543.00 (77.91%)	472.00 (67.72%)	414.00 (59.40%)	370.00 (53.08%)	
Yes	989.00 (35.47%)	154.00 (22.09%)	225.00 (32.28%)	283.00 (40.60%)	327.00 (46.92%)	
Renal dysfunction						<0.001
No	1,875.00 (67.25%)	586.00 (84.07%)	484.00 (69.44%)	438.00 (62.84%)	367.00 (52.65%)	
Yes	913.00 (32.75%)	111.00 (15.93%)	213.00 (30.56%)	259.00 (37.16%)	330.00 (47.35%)	
Leukemia						<0.001
No	2,332.00 (83.64%)	667.00 (95.70%)	608.00 (87.23%)	549.00 (78.77%)	508.00 (72.88%)	
Yes	456.00 (16.36%)	30.00 (4.30%)	89.00 (12.77%)	148.00 (21.23%)	189.00 (27.12%)	
Atrial fibrillation						0.02
No	2,053.00 (73.64%)	542.00 (77.76%)	506.00 (72.60%)	492.00 (70.59%)	513.00 (73.60%)	
Yes	735.00 (26.36%)	155.00 (22.24%)	191.00 (27.40%)	205.00 (29.41%)	184.00 (26.40%)	
Coronary artery disease						<0.001
No	2,019.00 (72.42%)	542.00 (77.76%)	529.00 (75.90%)	501.00 (71.88%)	447.00 (64.13%)	
Yes	769.00 (27.58%)	155.00 (22.24%)	168.00 (24.10%)	196.00 (28.12%)	250.00 (35.87%)	
Hypertension						0.002
No	1,427.00 (51.18%)	318.00 (45.62%)	353.00 (50.65%)	369.00 (52.94%)	387.00 (55.52%)	
Yes	1,361.00 (48.82%)	379.00 (54.38%)	344.00 (49.35%)	328.00 (47.06%)	310.00 (44.48%)	
Acute kidney injury						<0.001
No	1,882.00 (67.50%)	587.00 (84.22%)	486.00 (69.73%)	439.00 (62.98%)	370.00 (53.08%)	
Yes	906.00 (32.50%)	110.00 (15.78%)	211.00 (30.27%)	258.00 (37.02%)	327.00 (46.92%)	
Liver cirrhosis						<0.001
No	2,703.00 (96.95%)	693.00 (99.43%)	678.00 (97.27%)	664.00 (95.27%)	668.00 (95.84%)	
Yes	85.00 (3.05%)	4.00 (0.57%)	19.00 (2.73%)	33.00 (4.73%)	29.00 (4.16%)	
Hepatitis						0.048
No	2,715.00 (97.38%)	685.00 (98.28%)	675.00 (96.84%)	671.00 (96.27%)	684.00 (98.13%)	
Yes	73.00 (2.62%)	12.00 (1.72%)	22.00 (3.16%)	26.00 (3.73%)	13.00 (1.87%)	
Chronic kidney disease						<0.001
No	2,249.00 (80.67%)	606.00 (86.94%)	583.00 (83.64%)	557.00 (79.91%)	503.00 (72.17%)	
Yes	539.00 (19.33%)	91.00 (13.06%)	114.00 (16.36%)	140.00 (20.09%)	194.00 (27.83%)	
Malignancy						0.791
No	2,422.00 (86.87%)	609.00 (87.37%)	601.00 (86.23%)	601.00 (86.23%)	611.00 (87.66%)	
Yes	366.00 (13.13%)	88.00 (12.63%)	96.00 (13.77%)	96.00 (13.77%)	86.00 (12.34%)	
Hyperlipidemia						0.481
No	1,528.00 (54.81%)	369.00 (52.94%)	387.00 (55.52%)	396.00 (56.81%)	376.00 (53.95%)	
Yes	1,260.00 (45.19%)	328.00 (47.06%)	310.00 (44.48%)	301.00 (43.19%)	321.00 (46.05%)	
Heart failure						<0.001
No	2,023.00 (72.56%)	565.00 (81.06%)	512.00 (73.46%)	489.00 (70.16%)	457.00 (65.57%)	
Yes	765.00 (27.44%)	132.00 (18.94%)	185.00 (26.54%)	208.00 (29.84%)	240.00 (34.43%)	
Myocardial infarction						<0.001
No	2,495.00 (89.49%)	650.00 (93.26%)	626.00 (89.81%)	624.00 (89.53%)	595.00 (85.37%)	
Yes	293.00 (10.51%)	47.00 (6.74%)	71.00 (10.19%)	73.00 (10.47%)	102.00 (14.63%)	
Lschemic heart disease						<0.001
No	1,812.00 (64.99%)	502.00 (72.02%)	472.00 (67.72%)	438.00 (62.84%)	400.00 (57.39%)	
Yes	976.00 (35.01%)	195.00 (27.98%)	225.00 (32.28%)	259.00 (37.16%)	297.00 (42.61%)	
COPD						0.008
No	2,493.00 (89.42%)	645.00 (92.54%)	623.00 (89.38%)	619.00 (88.81%)	606.00 (86.94%)	
Yes	295.00 (10.58%)	52.00 (7.46%)	74.00 (10.62%)	78.00 (11.19%)	91.00 (13.06%)	
COVID-19						0.02
No	2,773.00 (99.46%)	697.00 (100.00%)	695.00 (99.71%)	692.00 (99.28%)	689.00 (98.85%)	
Yes	15.00 (0.54%)	0.00 (0.00%)	2.00 (0.29%)	5.00 (0.72%)	8.00 (1.15%)	
Laboratory result at 1st day						
Hematocrit, %	34.73 (29.69, 39.30)	36.40 (32.17, 40.85)	35.85 (30.80, 39.60)	33.50 (28.50, 38.75)	33.00 (28.13, 37.90)	<0.001
Hemoglobin, g/dL	11.40 (9.66, 13.00)	12.05 (10.50, 13.60)	11.90 (9.97, 13.20)	11.00 (9.35, 12.80)	10.60 (9.07, 12.50)	<0.001
RDW, %	14.13 (13.24, 15.40)	13.70 (13.00, 14.73)	14.04 (13.25, 15.20)	14.30 (13.28, 15.90)	14.50 (13.55, 15.85)	<0.001
Red blood cell count, ×10^12^/L	3.84 (3.26, 4.37)	4.03 (3.57, 4.51)	3.94 (3.37, 4.42)	3.69 (3.14, 4.29)	3.61 (3.09, 4.27)	<0.001
Anion gap, mmol/L	14.00 (12.00, 16.00)	13.00 (11.50, 15.00)	14.00 (12.00, 16.00)	14.00 (12.00, 16.00)	14.67 (12.50, 17.00)	<0.001
Total calcium, mmol/L	8.55 (8.10, 8.98)	8.70 (8.25, 9.05)	8.60 (8.15, 9.00)	8.45 (8.00, 8.90)	8.40 (7.98, 8.90)	<0.001
Chloride, mmol/L	104.67 (101.00, 108.00)	105.00 (102.00, 107.33)	104.50 (101.00, 107.67)	105.00 (101.50, 108.25)	104.00 (100.50, 108.00)	0.046
Potassium, mmol/L	4.05 (3.75, 4.43)	4.00 (3.70, 4.30)	4.05 (3.77, 4.40)	4.10 (3.80, 4.46)	4.08 (3.80, 4.55)	0.002
Sodium, mmol/L	139.00 (136.93, 142.00)	139.33 (137.00, 142.00)	139.40 (137.00, 141.60)	139.50 (136.50, 142.40)	139.00 (136.00, 142.00)	0.178
Magnesium, mmol/L	2.00 (1.83, 2.15)	2.00 (1.83, 2.10)	2.00 (1.83, 2.13)	2.00 (1.83, 2.20)	2.00 (1.82, 2.20)	0.316
Bicarbonate, mmol/L	22.50 (20.33, 24.50)	23.00 (21.33, 25.00)	23.00 (21.00, 24.50)	22.00 (20.00, 24.00)	22.00 (19.00, 24.25)	<0.001
Platelet count, ×10^9^/L	202.00 (152.00, 265.00)	213.00 (169.00, 266.50)	200.25 (154.50, 261.50)	194.00 (142.00, 258.50)	201.00 (143.67, 273.00)	<0.001
White blood cell count, ×10^9^/L	10.90 (8.30, 14.40)	10.00 (7.90, 12.70)	11.00 (8.60, 14.25)	11.30 (8.50, 15.00)	11.50 (8.40, 15.58)	<0.001
Glucose, mg/dL	130.50 (107.50, 165.67)	115.00 (100.00, 136.00)	126.67 (109.00, 147.00)	135.00 (109.60, 167.67)	166.00 (122.00, 211.75)	<0.001
INR	1.20 (1.10, 1.40)	1.15 (1.10, 1.30)	1.20 (1.10, 1.37)	1.20 (1.10, 1.43)	1.25 (1.10, 1.45)	<0.001
PTT, seconds	29.80 (26.50, 37.78)	29.40 (26.60, 37.67)	29.50 (26.40, 35.45)	29.76 (26.23, 37.83)	30.77 (26.65, 39.27)	0.023
MCHC, g/dL	32.87 (31.85, 33.80)	33.00 (32.10, 33.85)	33.03 (32.10, 33.90)	32.80 (31.70, 33.80)	32.65 (31.50, 33.70)	<0.001
PT, seconds	13.25 (12.10, 15.20)	12.70 (11.80, 14.20)	13.15 (12.05, 15.00)	13.53 (12.30, 15.60)	13.70 (12.30, 15.83)	<0.001
Creatinine, mg/dL	0.95 (0.75, 1.35)	0.90 (0.70, 1.10)	0.90 (0.70, 1.30)	1.00 (0.75, 1.43)	1.10 (0.80, 1.73)	<0.001
BUN, mg/dL	18.00 (12.50, 27.29)	15.50 (11.50, 21.33)	16.50 (12.00, 25.00)	19.00 (13.00, 29.43)	21.67 (14.67, 36.00)	<0.001
MCH, pg	30.00 (28.50, 31.36)	30.20 (28.80, 31.40)	30.10 (28.70, 31.40)	30.05 (28.45, 31.40)	29.78 (28.15, 31.05)	0.004
Procedure at 1st day, *n* (%)						
CRRT						<0.001
No	2,643.00 (94.80%)	693.00 (99.43%)	679.00 (97.42%)	652.00 (93.54%)	619.00 (88.81%)	
Yes	145.00 (5.20%)	4.00 (0.57%)	18.00 (2.58%)	45.00 (6.46%)	78.00 (11.19%)	
Mechanical ventilation						<0.001
No	629.00 (22.56%)	278.00 (39.89%)	146.00 (20.95%)	110.00 (15.78%)	95.00 (13.63%)	
Yes	2,159.00 (77.44%)	419.00 (60.11%)	551.00 (79.05%)	587.00 (84.22%)	602.00 (86.37%)	
Medication at 1st day, *n* (%)						
Antibiotics						<0.001
No	789.00 (28.30%)	348.00 (49.93%)	197.00 (28.26%)	138.00 (19.80%)	106.00 (15.21%)	
Yes	1,999.00 (71.70%)	349.00 (50.07%)	500.00 (71.74%)	559.00 (80.20%)	591.00 (84.79%)	
Nephrotoxic drugs						0.02
No	255.00 (9.15%)	83.00 (11.91%)	63.00 (9.04%)	58.00 (8.32%)	51.00 (7.32%)	
Yes	2,533.00 (90.85%)	614.00 (88.09%)	634.00 (90.96%)	639.00 (91.68%)	646.00 (92.68%)	
Glucocorticoids						<0.001
No	2,183.00 (78.30%)	606.00 (86.94%)	557.00 (79.91%)	522.00 (74.89%)	498.00 (71.45%)	
Yes	605.00 (21.70%)	91.00 (13.06%)	140.00 (20.09%)	175.00 (25.11%)	199.00 (28.55%)	
Vasopressors						<0.001
No	1,494.00 (53.59%)	523.00 (75.04%)	394.00 (56.53%)	325.00 (46.63%)	252.00 (36.15%)	
Yes	1,294.00 (46.41%)	174.00 (24.96%)	303.00 (43.47%)	372.00 (53.37%)	445.00 (63.85%)	
Beta blockers						<0.001
No	717.00 (25.72%)	238.00 (34.15%)	161.00 (23.10%)	164.00 (23.53%)	154.00 (22.09%)	
Yes	2,071.00 (74.28%)	459.00 (65.85%)	536.00 (76.90%)	533.00 (76.47%)	543.00 (77.91%)	
Statins						0.85
No	2,256.00 (80.92%)	563.00 (80.77%)	558.00 (80.06%)	571.00 (81.92%)	564.00 (80.92%)	
Yes	532.00 (19.08%)	134.00 (19.23%)	139.00 (19.94%)	126.00 (18.08%)	133.00 (19.08%)	
Antiplatelet agents						0.688
No	710.00 (25.47%)	177.00 (25.39%)	171.00 (24.53%)	189.00 (27.12%)	173.00 (24.82%)	
Yes	2,078.00 (74.53%)	520.00 (74.61%)	526.00 (75.47%)	508.00 (72.88%)	524.00 (75.18%)	
Insulin						<0.001
No	655.00 (23.49%)	229.00 (32.86%)	196.00 (28.12%)	152.00 (21.81%)	78.00 (11.19%)	
Yes	2,133.00 (76.51%)	468.00 (67.14%)	501.00 (71.88%)	545.00 (78.19%)	619.00 (88.81%)	
Antihypertensive drugs						<0.001
No	771.00 (27.65%)	279.00 (40.03%)	181.00 (25.97%)	166.00 (23.82%)	145.00 (20.80%)	
Yes	2,017.00 (72.35%)	418.00 (59.97%)	516.00 (74.03%)	531.00 (76.18%)	552.00 (79.20%)	
Prognosis, *n* (%)						
Hospital day	10.92 (6.25, 19.75)	7.70 (4.83, 13.06)	11.28 (6.49, 19.26)	13.03 (7.80, 22.86)	13.15 (7.20, 23.63)	<0.001
ICU day	4.76 (2.75, 8.74)	3.60 (2.33, 5.60)	4.96 (2.77, 8.54)	5.61 (3.06, 10.00)	5.60 (2.82, 11.19)	<0.001
All-cause mortality						<0.001
No	1,492.00 (53.52%)	457.00 (65.57%)	403.00 (57.82%)	356.00 (51.08%)	276.00 (39.60%)	
Yes	1,296.00 (46.48%)	240.00 (34.43%)	294.00 (42.18%)	341.00 (48.92%)	421.00 (60.40%)	
In-hospital mortality						0.069
No	2,582.00 (92.61%)	654.00 (93.83%)	648.00 (92.97%)	650.00 (93.26%)	630.00 (90.39%)	
Yes	206.00 (7.39%)	43.00 (6.17%)	49.00 (7.03%)	47.00 (6.74%)	67.00 (9.61%)	
ICU mortality						<0.001
No	2,306.00 (82.71%)	638.00 (91.54%)	595.00 (85.37%)	564.00 (80.92%)	509.00 (73.03%)	
Yes	482.00 (17.29%)	59.00 (8.46%)	102.00 (14.63%)	133.00 (19.08%)	188.00 (26.97%)	
28-day all-cause mortality						<0.001
No	2,298.00 (82.42%)	619.00 (88.81%)	584.00 (83.79%)	573.00 (82.21%)	522.00 (74.89%)	
Yes	490.00 (17.58%)	78.00 (11.19%)	113.00 (16.21%)	124.00 (17.79%)	175.00 (25.11%)	
90-day all-cause mortality						<0.001
No	2,237.00 (80.24%)	614.00 (88.09%)	571.00 (81.92%)	558.00 (80.06%)	494.00 (70.88%)	
Yes	551.00 (19.76%)	83.00 (11.91%)	126.00 (18.08%)	139.00 (19.94%)	203.00 (29.12%)	
180-day all-cause mortality						<0.001
No	2,224.00 (79.77%))	612.00 (87.80%)	570.00 (81.78%)	554.00 (79.48%)	488.00 (70.01%)	
Yes	564.00 (20.23%)	85.00 (12.20%)	127.00 (18.22%)	143.00 (20.52%)	209.00 (29.99%)	
365-day all-cause mortality						<0.001
No	2,196.00 (78.77%)	608.00 (87.23%)	565.00 (81.06%)	549.00 (78.77%)	474.00 (68.01%)	
Yes	592.00 (21.23%)	89.00 (12.77%)	132.00 (18.94%)	148.00 (21.23%)	223.00 (31.99%)	

Q1: GV ≤ 10.8%; Q2: 10.8% < GV ≤ 16.27%; Q3:16.27% < GV ≤ 24.07%; Q4: GV > 24.07%.

Although the validation cohort was smaller and differed in some demographic features (likely due to its limited sample size), it showed similar trends in core clinical outcomes (e.g., disease severity scores, all-cause mortality) as the original cohort. This qualitative consistency provides preliminary support for the direction of the association observed in the original cohort.

### 3.2 Variable screening and feature importance analysis

**Figure 3:**
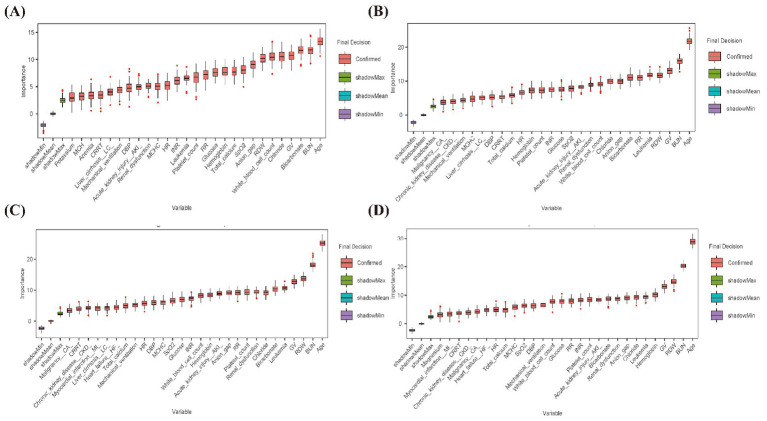
The Boruta algorithm shows the relative importance of predictors for ACM at four time points: **(A)** 28 days, **(B)** 90 days, **(C)** 180 days, and **(D)** 365 days. GV consistently ranked among the top five predictors across all time periods, underscoring its clinical relevance over both short- and long-term horizons.

The Boruta algorithm consistently identified GV as a top-tier predictor of mortality across all time points, placing it within the top five most important variables in each analysis (Figure 3). This indicates its consistent and sustained association with prognosis across different time points. Supporting this finding, LASSO regression consistently selected GV for inclusion in all prediction models (28- to 365-day), with the magnitude of its regression coefficient increasing over time (Supplementary Figures S4, S5, Supplementary Table S4). This pattern suggests that GV’s influence on mortality risk grows stronger in the long term. Integrating results from both methods supports GV as a stable factor associated with mortality across all time periods. We also observed a shift in predictor importance over time. As time progressed, the influence of acute physiological markers (e.g., DBP, Acute kidney injury) gradually declined, while variables reflecting chronic organ dysfunction (e.g., CKD, heart failure, malignancy, and BUN) became relatively more significant.

### 3.3 Survival analysis based on GV quartile grouping

**Figure 4:**
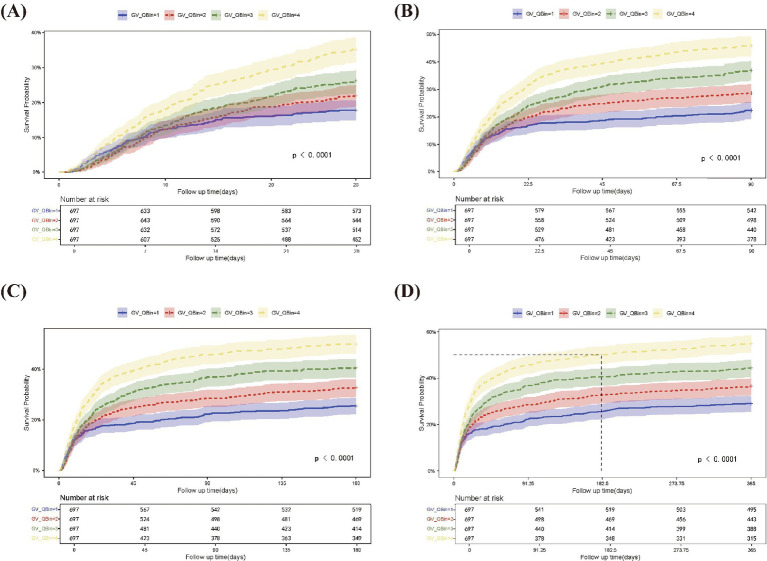
Kaplan–Meier survival curves in original cohort show the probability of ACM across GV quartiles (Q1–Q4) at four time points after ICU admission: **(A)** 28 days, **(B)** 90 days, **(C)** 180 days, and **(D)** 365 days.

**Figure 5:**
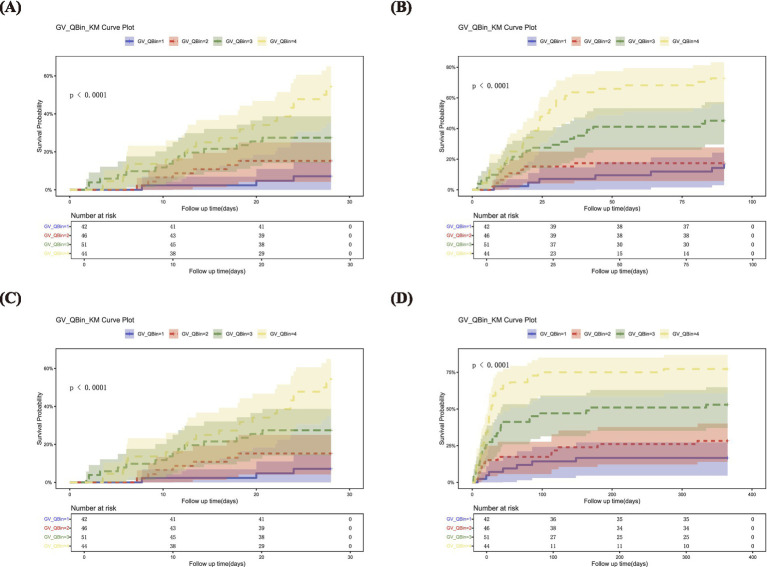
Kaplan–Meier survival curves in validation cohort show the probability of ACM across GV quartiles (Q1–Q4) at four time points after ICU admission: **(A)** 28 days, **(B)** 90 days, **(C)** 180 days, and **(D)** 365 days.

In both the original and validation cohort, K–M survival curves exhibited distinct separation among the GV quartiles, indicating distinct survival probabilities (log-rank test, all *p* < 0.0001; Figures 4, 5). Survival rates declined sharply with higher GV levels, and this pattern remained consistently across all time points (28 to 365 days). The survival curve for the highest GV quartile (Q4) showed a sharply downward at an early stage.

### 3.4 Determination and validation of optimal GV risk stratification thresholds

We performed exploratory analyses to identify potential thresholds of GV for risk stratification. As Supplementary Table S5 shows, the optimal cutoff values decreased gradually as follow-up time increased. Considering both data variability and clinical applicability, we explored GV thresholds of approximately 30% for 28-day, 20% for 90/180-day, and 15% for 365-day mortality stratification (Supplementary Table S6). After stratifying patients into high- and low-GV groups according to these thresholds, the high-GV group showed a significantly higher risk of ACM at all follow-up time points: 28 days, HR = 1.411 (95% CI: 1.170–1.701); 90 days, HR = 1.298 (95% CI: 1.129–1.492); 180 days, HR = 1.256 (95% CI: 1.101–1.434); and 365 days, HR = 1.290 (95% CI: 1.133–1.468); all *p* < 0.001. To explore the discriminatory performance of these cutoff values, K-M survival curves were generated according to this threshold (Supplementary Figures S6, S7). At each of the four follow-up assessments (28, 90, 180, and 365 days), the high-GV group demonstrated significantly reduced survival probabilities compared to the low-GV group (log-rank test, all *p* < 0.0001). These exploratory, data-derived thresholds showed qualitative concordance between the validation and original cohorts, suggesting a potential time-dependent pattern: in short-term outcomes, only markedly elevated GV levels (>30%) were associated with increased mortality risk, whereas in long-term follow-up, even mild elevations (>15%) appeared sufficient to differentiate high-risk patients.

Supplementary Figure S8 shows the results of the Spearman’s correlation analysis. Because of strong correlations among certain variables—including SBP and DBP; BUN and creatinine (*ρ* = 0.95); red blood cell count, hematocrit, and hemoglobin (ρ ≥ 0.98); INR and PT (ρ = 1); sodium and chloride (ρ = 0.77); and multiple severity scores (SOFA, SAPS II, OASIS, APS III, and APACHE II, ρ ≥ 0.8). These parameters were excluded from subsequent modeling to prevent multicollinearity and potential bias in model estimation.

### 3.5 Cox proportional hazards regression analysis

Table 2 shows, GV levels demonstrated a significant positive trend with ACM in both Model 1 and Model 2 (P for trend < 0.001). The higher GV quartiles (Q3: 16.27% < GV ≤ 24.07%; Q4: GV > 24.07%) exhibited significantly higher mortality risks compared with the Q1 group (all *p* ≤ 0.001). In Model 3, the association of GV with ACM remained statistically significant. Compared with the Q1 group, the Q4 group showed elevated ACM risks of 1.307 (95% CI: 1.033–1.654, *p* = 0.026), 1.473 (95% CI: 1.196–1.814, *p* < 0.001), 1.447 (95% CI: 1.190–1.760, *p* < 0.001), and 1.486 (95% CI: 1.236–1.788, *p* < 0.001) at 28, 90, 180, and 365 days, respectively. As the model underwent progressive adjustments, the risk ratio for the Q4 group decreased slightly but remained statistically significant (*p* < 0.05), whereas the Q2 and Q3 groups showed no significant associations in Model 3 (*p* > 0.05). Patients with high GV showed a significant increase in long-term ACM after ICU admission compared to those with low GV, with this relationship maintaining significance after multilevel adjustments.

**Table 2:** Multivariable Cox regression analyses of GV and ACM at different follow-up time points.

Outcome	Model 1			Model 2			Model 3		
	HR (95%CI)	*P*	*P* for trend	HR (95%CI)	*P*	*P* for trend	HR (95%CI)	*P*	*P* for trend
Original cohort									
28-day ACM									
Q1	Reference			Reference			Reference		
Q2	1.238 (0.977, 1.569)	0.077	<0.001	1.190 (0.939, 1.508)	0.150	<0.001	0.950 (0.746, 1.209)	0.677	<0.001
Q3	1.511 (1.203, 1.897)	<0.001		1.468 (1.168, 1.844)	0.001		1.013 (0.798, 1.286)	0.916	
Q4	2.143 (1.726, 2.659)	<0.001		2.098 (1.689, 2.606)	<0.001		1.307 (1.033, 1.654)	0.026	
90-day ACM									
Q1	Reference								
Q2	1.314 (1.065, 1.62 1)	0.011	<0.001	1.259 (1.021, 1.554)	0.031	<0.001	1.020 (0.824, 1.264)	0.853	<0.001
Q3	1.760 (1.442, 2.149)	<0.001		1.712 (1.402, 2.09)	<0.001		1.194 (0.969, 1.471)	0.096	
Q4	2.361 (1.948, 2.861)	<0.001		2.321 (1.915, 2.814)	<0.001		1.473 (1.196, 1.814)	<0.001	
180-day ACM									
Q1	Reference								
Q2	1.321 (1.086, 1.607)	0.005	<0.001	1.264 (1.039, 1.538)	0.019	<0.001	1.022 (0.837, 1.248)	0.828	<0.001
Q3	1.718 (1.424, 2.072)	<0.001		1.673 (1.387, 2.019)	<0.001		1.173 (0.964, 1.427)	0.110	
Q4	2.301 (1.920, 2.757)	<0.001		2.269 (1.892, 2.719)	<0.001		1.447 (1.190, 1.760)	<0.001	
365-day ACM									
Q1	Reference								
Q2	1.306 (1.086, 1.571)	0.005	<0.001	1.248 (1.038, 1.502)	0.019	<0.001	1.017 (0.843, 1.228)	0.857	<0.001
Q3	1.678 (1.405, 2.004)	<0.001		1.637 (1.370, 1.955)	<0.001		1.171 (0.973, 1.409)	0.095	
Q4	2.281 (1.923, 2.706)	<0.001		2.256 (1.901, 2.677)	<0.001		1.486 (1.236, 1.788)	<0.001	
Validation cohort									
28-day ACM									
Q1	Reference			Reference			Reference		
Q2	2.280 (0.589, 8.817)	0.232	<0.001	2.191 (0.565, 8.495)	0.257	<0.001	1.758 (0.414, 7.460)	0.444	<0.001
Q3	4.576 (1.315, 15.927)	0.017		4.403 (1.260, 15.394)	0.020		3.804 (1.015, 14.247)	0.047	
Q4	9.791 (2.945, 32.547)	<0.001		9.458 (2.837, 31.535)	<0.001		7.729 (2.091, 28.575)	0.002	
90-day ACM									
Q1	Reference								
Q2	1.125 (0.408, 3.104)	0.819	<0.001	1.060 (0.383, 2.931)	0.910	<0.001	0.769 (0.255, 2.324)	0.642	<0.001
Q3	3.480 (1.493, 8.115)	0.004		3.302 (1.410, 7.73)	0.006		2.612 (1.048, 6.514)	0.039	
Q4	6.998 (3.075, 15.926)	<0.001		6.669 (2.923, 15.213)	<0.001		5.19 (2.045, 13.170)	0.001	
180-day ACM									
Q1	Reference								
Q2	1.688 (0.665, 4.289)	0.271	<0.001	1.553 (0.610, 3.954)	0.355	<0.001	1.110 (0.401, 3.074)	0.840	<0.001
Q3	4.048 (1.756, 9.331)	0.001		3.768 (1.629, 8.716)	0.002		3.066 (1.252, 7.509)	0.014	
Q4	7.776 (3.423, 17.669)	<0.001		7.255 (3.187, 16.515)	<0.001		5.518 (2.198, 13.85)	<0.001	
365-day ACM									
Q1	Reference								
Q2	1.834 (0.732, 4.597)	0.196	<0.001	1.687 (0.672, 4.237)	0.266	<0.001	1.142 (0.420, 3.100)	0.795	<0.001
Q3	4.253 (1.851, 9.772)	0.001		3.968 (1.722, 9.147)	0.001		3.101 (1.274, 7.553)	0.013	
Q4	8.234 (3.631, 18.672)	<0.001		7.632 (3.359, 17.338)	<0.01		5.613 (2.251, 13.995)	<0.001	

Model 1 was unadjusted; Model 2 was adjusted for age, sex, race; Model 3 was Model 2 further adjusted for vital signs (HR, RR, DBP), severity scores (SOFA, GCS), comorbidities (CKD, HF, MI, malignancy, COPD), medications (Vasopressors, Beta-blockers, Statins, Antiplatelet agents), and procedures (CRRT and MV). Abbreviations are as in Table 1. The hazard ratios in the validation cohort, while demonstrating a consistent positive association, are derived from a cohort of limited sample size (*n* = 183) and should be interpreted as preliminary evidence of effect direction.

In the validation cohort, a positive association between high GV and ACM was observed, supporting the direction of the association found in the original cohort. Under the same fully adjusted model (Model 3), the Q4 group showed increased mortality risk at 28, 90, 180, and 365 days, with HRs of 7.729, 5.190, 5.518, and 5.613, respectively (all *p* < 0.001). The number of mortality events in the validation cohort at these time points were *n* = 48, *n* = 70, *n* = 78, and *n* = 81, out of 183 patients, as detailed in the Supplementary Table S3. Due to the small sample size of the validation cohort (*n* = 183), the point estimates of these hazard ratios—particularly those with exceptionally high values and wide confidence intervals—are statistically unstable. Therefore, these results should be viewed primarily as confirming the direction of the association observed in the derivation cohort, rather than providing a precise estimate of its magnitude.

### 3.6 Nonlinear and threshold effect analysis of GV

**Figure 6:**
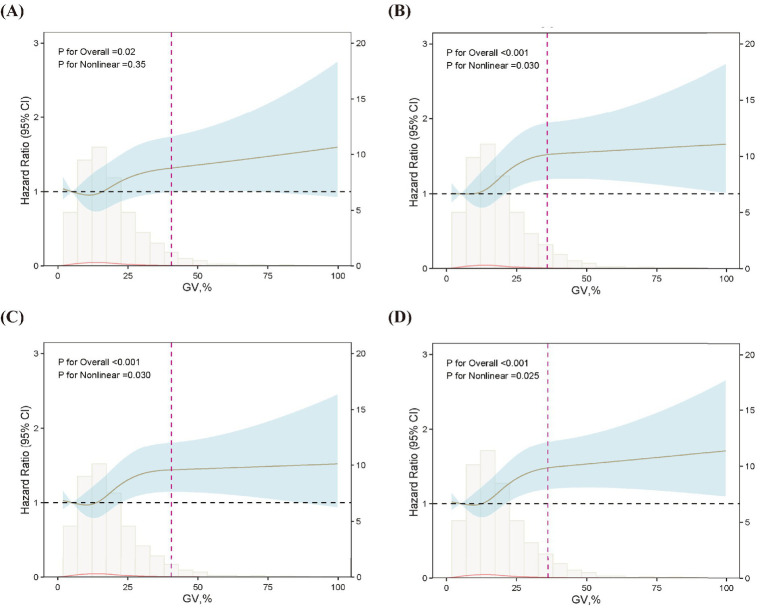
Nonlinear association between GV and ACM assessed by restricted cubic splines in original cohort. The plots show the hazard ratios (95% CI) for mortality in relation to GV levels at **(A)** 28, **(B)** 90, **(C)** 180, and **(D)** 365 days.

**Figure 7:**
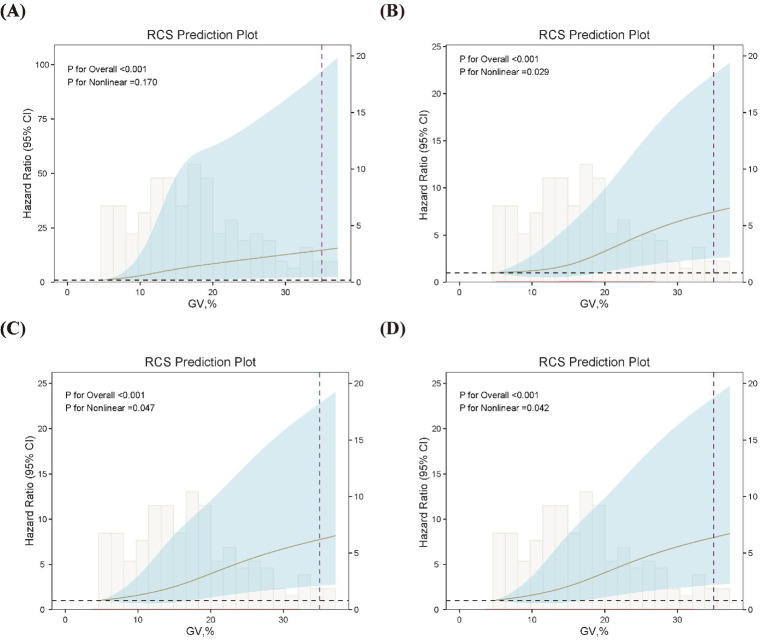
Nonlinear association between GV and ACM assessed by restricted cubic splines in validation cohort. The plots show the hazard ratios (95% CI) for mortality in relation to GV levels at **(A)** 28, **(B)** 90, **(C)** 180, and **(D)** 365 days.

RCS analysis (Figures 6, 7) demonstrated a significant positive relationship between GV and ACM at all time points (*P* for overall = 0.02 at 28 days; *P* for overall < 0.001 at 90, 180, and 365 days). Significant nonlinearity was detected in the 90-, 180-, and 365-days models (*P* for nonlinearity = 0.030, 0.030, and 0.025, respectively). The curve suggested that mortality risk was relatively stable at low GV levels, increased more steeply beyond approximately 16–18%, and then appeared to plateau at higher levels around 36–38%.

To further explore this nonlinear effect, a threshold effect analysis was performed using a two-stage Cox proportional hazards model (Table 3). The results suggested a potential inflection point near 36%. Two-stage piecewise model consistently outperformed the simple linear model in log-likelihood ratio tests (*p* < 0.01). Below this point, the risk increased with GV; above it, the increasing trend was attenuated. It is important to note that this observed plateau could represent a true biological saturation effect, or alternatively, reflect limited data density and higher variability in risk estimation at the highest GV levels.

**Table 3:** Threshold effect analysis of GV on ACM using a two-piecewise Cox proportional hazards model.

Analysis Item	Adjusted HR	*p*-value
Original cohort		
90-day ACM		
Fitting model by the standard Cox proportional hazards model	1.0079 (1.0039, 1.0120)	0.0001
Fitting model by the two-piecewise Cox proportional hazards model		
Inflection point	36.0400	
GV < 36.04%	1.0181 (1.0101, 1.0263)	<0.0001
GV > 36.04%	0.9990 (0.9909, 1.0073)	0.8200
*P*		0.0038
180-day ACM		
Fitting model by the standard Cox proportional hazards model	1.0073 (1.0034, 1.0113)	0.0002
Fitting model by the two-piecewise Cox proportional hazards model		
Inflection point	37.1400	
GV < 37.14%	1.0164 (1.0090, 1.0239)	<0.0001
GV > 37.14%	0.9983 (0.9901, 1.0066)	0.6897
*P*		0.0043
365-day ACM		
Fitting model by the standard Cox proportional hazards model	1.0084 (1.0048, 1.0121)	<0.0001
Fitting model by the two-piecewise Cox proportional hazards model		
Inflection point	36.2200	
GV < 36.22%	1.0175 (1.0103, 1.0247)	<0.0001
GV > 36.22%	1.0003 (0.9930, 1.0077)	0.9320
*P*		0.0039
Validation cohort		
90-day ACM		
Fitting model by the standard Cox proportional hazards model	1.0372 (1.0240, 1.0506)	<0.0001
Fitting model by the two-piecewise Cox proportional hazards model	36.0400	
Inflection point		
GV < 36.04%	1.0748 (1.0417, 1.1090)	<0.0001
GV > 36.04%	1.0218 (1.0016, 1.0424)	0.0341
*P*	0.0132	
180-day ACM		
Fitting model by the standard Cox proportional hazards model	1.0358 (1.0230, 1.0488)	<0.0001
Fitting model by the two-piecewise Cox proportional hazards model	37.1400	
Inflection point		
GV < 37.14%	1.0681 (1.0371, 1.1001)	<0.0001
GV > 37.14%	1.0206 (1.0001, 1.0416)	0.0490
*P*	0.0226	
365-day ACM		
Fitting model by the standard Cox proportional hazards model	1.0344 (1.0219, 1.0471)	<0.0001
Fitting model by the two-piecewise Cox proportional hazards model	36.2200	
Inflection point		
GV < 36.22%	1.0701 (1.0385, 1.1026)	<0.0001
GV > 36.22%	1.0192 (0.9995, 1.0393)	0.0564
*P*	0.0139	

The inflection points of GV for mortality risk were identified at 90, 180, and 365 days. HR and 95% CI are presented for GV values below and above each inflection point.

In contrast, while risk plateaued beyond the inflection point in the original cohort, the validation cohort exhibited a modest upward trend after this threshold, though this trend was not statistically significant. This discrepancy may stem from the limited number of cases with very high GV (>36%) in the validation cohort, reducing the precision of risk estimation within this plateau phase. Collectively, these nonlinear and threshold analyses are exploratory and hypothesis-generating. The specific cutoff and inflection points identified are derived from this specific cohort and require validation in future prospective studies before any clinical application can be considered.

### 3.7 Subgroup analysis

**Figure 8:**
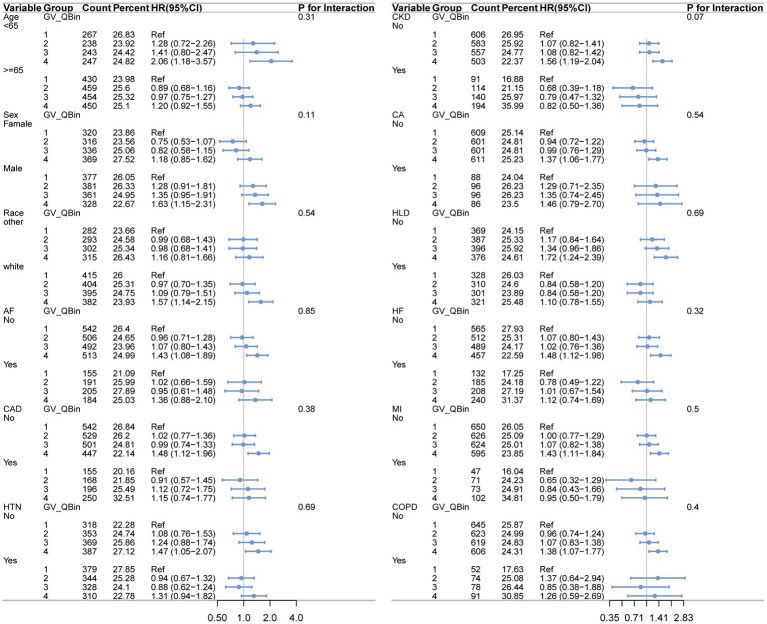
Subgroup analyses of the association of GV with 28-day ACM. No subgroup interactions proved statistically significant (all *P* for interaction > 0.05).

**Figure 9:**
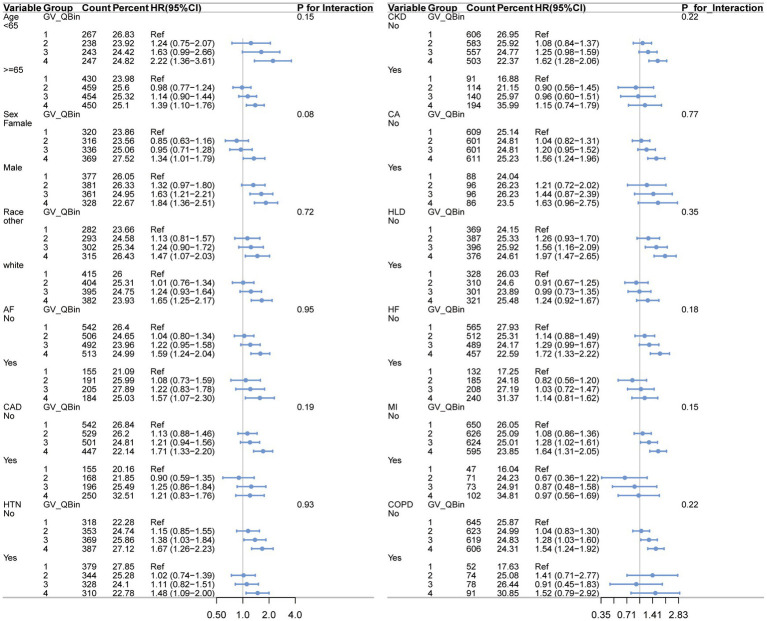
Subgroup analyses of the association of GV with 90-day ACM. No subgroup interactions proved statistically significant (all *P* for interaction > 0.05).

**Figure 10:**
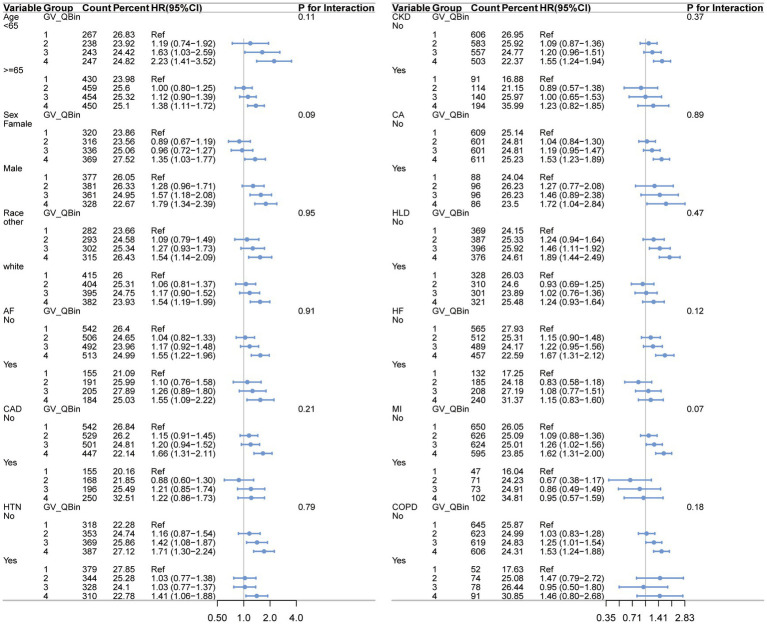
Subgroup analyses of the association of GV with 180-day ACM. No subgroup interactions proved statistically significant (all *P* for interaction > 0.05).

**Figure 11:**
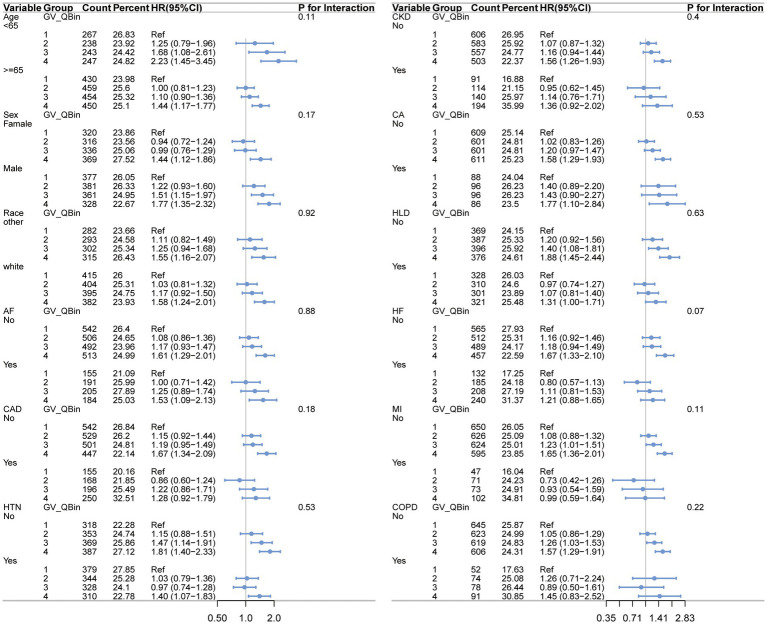
Subgroup analyses of the association of GV with 365-day ACM. No subgroup interactions proved statistically significant (all *P* for interaction > 0.05).

An evaluation of the consistency and robustness of the association between GV and ACM was conducted through subgroup analyses according to age, sex, ethnicity, and major comorbidities (Figure 8–11; Supplementary Tables S7–10). The results indicated that the predictive effect of GV was not significantly modified by all these factors (all interaction *p* ≥ 0.05).

Notably, exploratory observations within subgroups revealed variations in the strength and significance of the association for the high GV (Q4 group). While the direction of association was consistently positive, the hazard ratios for Q4 varied across subgroups. For instance, younger patientsm (<65 years) faced a numerically higher 28-day ACM risk (HR 2.06, 95% CI 1.18–3.57) compared with older patients (≥65 years, HR 1.20, 95% CI 0.92–1.55). Similarly, the point estimate for the association was numerically greater in males (90-day HR 1.84, 95% CI 1.36–2.51) compared with females (90-day HR 1.34, 95% CI 1.01–1.79), although the interaction *p*-value was 0.083. Furthermore, the association for Q4 reached statistical significance (*p* ≤ 0.05) in most, but not all, subgroups. For example, at 365 days, the Q4 association was significant in patients without heart failure (HR = 1.67, 95% CI 1.33–2.10, *p* ≤ 0.001) but was attenuated and non-significant in those with heart failure (HR = 1.21, 95% CI 0.88–1.65, *p* = 0.237). A comparable pattern was observed for other comorbidities such as coronary artery disease and chronic kidney disease.

These patterns suggest that the risk linked to high GV may vary between patients. The association appeared more pronounced in subgroups without major pre-existing comorbidities. This could indicate that GV’s role is easier to detect when the patient’s baseline risk is lower. However, as the interaction tests were not significant, these findings remain exploratory and require confirmation in future studies.

### 3.8 Predictive performance and incremental effect analysis of GV

**Figure 12:**
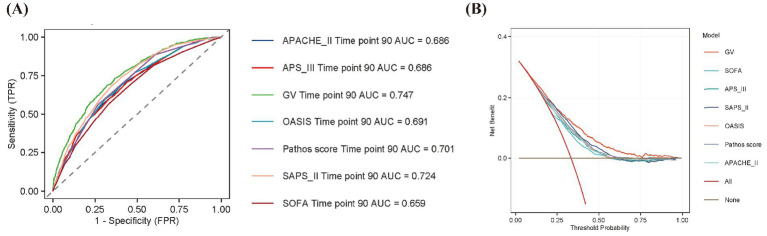
Predictive performance assessments evaluation using ROC curves and DCA. **(A)** ROC curves compare the discriminative ability of GV-based models against traditional severity scores. **(B)** DCA demonstrates the net clinical benefit of incorporating GV into risk stratification.

To further validate the clinical utility of GV in prognostic evaluation, predictive performance and incremental effect analyses were performed using the GV-based model. Based on both statistical and clinical considerations, we chose 90-day ACM as the representative endpoint for prognostic evaluation. The GV-based model’s ROC analysis (Figure 12) achieved an AUC of 0.747, significantly outperforming those of all traditional disease severity scores (SOFA: 0.659; APACHE II: 0.686; APS III: 0.686; OASIS: 0.691; Pathos score: 0.701; SAPS II: 0.724; all *p* < 0.05 by DeLong test), indicating superior discriminative ability. In clinical decision analysis, DCA (Figure 12) revealed the GV-based model yielded the greatest net clinical benefit across the commonly applied risk threshold range (0.2–0.7). Compared with traditional scoring models, the GV-based model offered greater practical value. In IDI and NRI, incorporating GV significantly enhanced the model’s reclassification and discrimination capabilities. Across ACM outcomes at four follow-up time points, the GV-based model exhibited statistically significant favorable Net NRI and Integrated IDI values (*p* < 0.05) (Supplementary Table S11): 28 days (NRI = 0.099, IDI = 0.003), 90 days (NRI = 0.131, IDI = 0.007), 180 days (NRI = 0.131, IDI = 0.006), and 365 days (NRI = 0.136, IDI = 0.008). Both NRI and IDI values showed a gradual upward trend over time, indicating that GV contributed progressively greater incremental value in predicting long-term mortality risk.

### 3.9 Sensitivity analysis

1. Analysis Excluding Patients Who Experienced Early Mortality

To reduce potential reverse causality, the analysis excluded patients who died within the initial 72 h following ICU admission. As shown in Supplementary Table S12, in the original cohort, the risk estimates for the high GV group (Q4, GV > 24.07%) were slightly higher than those in the main analysis: 28-day HR = 1.517 (95% CI, 1.114–2.066; *p* = 0.008), 90-day HR = 1.646 (95% CI, 1.258–2.154; *p* < 0.001), 180-day HR = 1.643 (95% CI, 1.273–2.121; *p* < 0.001), and 365-day HR = 1.690 (95% CI, 1.327–2.151; *p* < 0.001). A consistent direction of association was observed in the validation cohort. After accounting for early mortality, the link between high GV and ACM not only remained significant but also showed a stronger association.

1. Stratified Analysis by ICU Length of Stay

When stratified by ICU length of stay (≤3 vs. >3 days), the positive association between GV and ACM risk remained consistent across all time points (Supplementary Table S13). The association was more pronounced among patients with ICU stays longer than three days (P for interaction = 0.039–0.121), with HRs of 1.55 (95% CI, 1.41–2.12; *p* = 0.005) at 28 days, 1.69 (95% CI, 1.29–2.21; *p* < 0.001) at 90 days, 1.56 (95% CI, 1.21–2.03; *p* = 0.001) at 180 days, and 1.73 (95% CI, 1.36–2.20; *p* < 0.001) at 365 days. No significant association was observed between GV and ACM risk among patients with ICU stays of ≤3 days (*p* > 0.05). These results indicate that ICU length of stay may be an important modifier of the GV-mortality relationship, and patients with prolonged hospitalization accumulate a more pronounced risk.

1. Analysis of Adjusted Blood Glucose Sampling Parameters

As shown in Supplementary Table S14, in the original cohort, the direction of association between GV and ACM risk remained consistent after adjusting the total number of glucose measurements, mean sampling interval, and total sampling duration. Although the effect sizes were modestly reduced, the associations remained statistically significant at all time points: 28-day HR = 1.292 (95% CI, 1.004–1.663; *p* = 0.046), 90-day HR = 1.346 (95% CI, 1.076–1.684; *p* = 0.009), 180-day HR = 1.312 (95% CI, 1.063–1.620; *p* = 0.012), and 365-day HR = 1.336 (95% CI, 1.095–1.630; *p* = 0.004). A consistent direction of association was observed in the validation cohort. Results maintained stable under diverse sampling scenarios, demonstrating the stability of the observed associations.

1. Outlier Analysis

The positive association of GV and ACM risk remained consistent after limiting GV values to the 0th–96th percentile range and regrouping the data (Supplementary Table S15). Although the statistical significance for the 28-day outcome slightly decreased (HR = 1.257; 95% CI, 0.985–1.603; *p* = 0.066), mortality risks at 90, 180, and 365 days remained significantly elevated (all *p* < 0.001). These results confirm that the relationship between GV and long-term ACM risk was not significantly affected by outliers.

## 4 Discussion

This multicenter cohort study included data from MIMIC-IV and a validation cohort from Affiliated Hospital of North China University of Science and Technology (total *n* = 2,971). We systematically studied the association of GV with ACM in critically ill patients with ND-IS using data from the MIMIC-IV database. Elevated GV was consistently linked to higher short- and long-term ACM risks, even after adjusting for demographics, vital signs, severity scores, comorbidities, and treatments. Both K-M curves and multivariate Cox models showed significantly reduced survival at 28, 90, 180, and 365 days in patients with high GV. These results indicate that GV, as a composite indicator reflecting the fluctuation of metabolic homeostasis, is consistently and independently associated with prognosis in this population.

The RCS analysis indicated a nonlinear, positive association of GV with the risk of ACM. As GV exceeded approximately 36%, the mortality trend appeared to plateau, suggesting a potential saturation effect. Subsequent threshold effect analysis supported this nonlinear pattern, identifying a significant inflection point near 36–38%. Below this threshold, each GV increase significantly raised mortality risk, while beyond it, further GV changes showed no statistically meaningful impact. These exploratory findings suggest that the association between GV and mortality may follow a threshold-like pattern. Within a specific range, the cumulative metabolic burden induced by blood glucose fluctuations appears most pronounced. One plausible, though speculative, explanation is that beyond this range (36–38%), the body’s metabolic homeostasis may reach a state of relative exhaustion, where additional fluctuations contribute less to incremental risk.

Yang et al. (6) reported a significant U-shaped association between GV and ACM among patients with acute cerebral infarction, with effects persisting at both 90 days and 365 days. In contrast, GV exhibited a more stable association with ACM in intracerebral hemorrhage cases (25). These findings suggest that stroke subtype pathophysiology may influence susceptibility to glycemic fluctuations and their prognostic implications. Our study further confirms the long-term prognostic value of GV in patients with IS and highlights disease-specific patterns in metabolic response.

We used Boruta and LASSO regression to identify key predictive variables for outcomes across multiple time points, assessing GV’s stability and long-term prognostic value. GV emerged as a consistent core predictor in all models (28 to 365 days), with its relative importance increasing throughout the follow-up period. In the early-stage (28-day) models, GV showed importance comparable to acute physiological markers (e.g., HR, SOFA score, and CRRT), reflecting an acute stress-induced metabolic imbalance. During the intermediate period (90 and 180 days), the relative weight of GV increased, ranking alongside chronic organ function indicators such as BUN, CKD, and HF. In the long-term (365-day) model, GV reached peak importance, emerging as one of most important predictors to long-term mortality. This trend suggests that mortality determinants in critically ill stroke patients transition from acute physiological dependence to chronic metabolic dependence as the disease progresses. GV serve as a stable metabolic biomarker across this clinical progression. Building upon these findings, we propose a time-dependent risk migration model (a form of dynamic risk evolution). This model describes how GV’s risk contribution progressively shifts from acute metabolic fluctuations to chronic metabolic exhaustion as the disease advances. It thereby offers a plausible explanation for the observed systemic metabolic evolution in critically ill stroke patients.

The stronger association of GV with long-term mortality could reflect evolving pathophysiological contributions over time (26, 27). In the acute phase, marked glucose fluctuations could induce oxidative stress and inflammatory responses, exacerbating initial brain injury (25, 28–30). Over the long term, persistent GV might contribute to a state of chronic metabolic dysregulation and endothelial dysfunction, potentially leading to progressive organ damage (28, 31–33). These proposed mechanisms, drawn from the broader literature on dysglycemia, provide a plausible, though speculative, framework for interpreting the observed time-dependent strengthening of the association. However, they remain speculative within the context of our present study, which was not designed to test these specific pathways.

Previous research has demonstrated that elevated GV consistently correlates with adverse outcomes in hospitalized and critically ill patients, including increased short-term mortality. This association remains significant after accounting for mean glucose levels and hypoglycemia, appearing across different GV metrics like coefficient of variation (CV), standard deviation (SD), and glycemic load index (GLI) (11, 26, 34). In acute stroke populations, a meta-analysis of cohort studies linked high GV during hospitalization or the perioperative period to early mortality and poor neurological outcomes (11). Another continuous glucose monitoring (CGM) study similarly found that raised GV predicted adverse outcomes, including mortality, during both hospitalization and follow-up. The effect was especially strong in patients with large vessel occlusion acute ischemic stroke who received mechanical thrombectomy, where higher GV markedly increased in-hospital and 90-day mortality (35). Multicenter studies have established GV as a dynamic marker of metabolic stress in critical illness and have reported it to be independently associated with short-term mortality (8, 34). Expanding on this work, our analysis extends the follow-up period and focuses specifically on ND-IS patients in the ICU. This selective approach minimized confounding from pre-existing glucose metabolism disorders. Our results show GV independently predicts 28-day mortality and exhibits a time-dependent strengthening effect on mortality risk at 90, 180, and 365 days.

Current international guidelines, including the ADA 2025 Hospital Care (36), SCCM 2024 ICU Glycemic Management (37), and AHA/ASA 2024 Acute Ischemic Stroke Guideline (38), focus on maintaining blood glucose between 140 and 180 mg/dL and preventing hypoglycemia in ICU patients. However, they lack specific recommendations for monitoring or managing GV. Our work draws attention to this gap by providing evidence that GV is independently associated with mortality risk in critically ill non-diabetic populations. We further show that GV’s impact has a time-dependent amplification on long-term mortality risk. These results suggest that incorporating GV stabilization into routine management could add substantial clinical value beyond current glucose management targets. This approach supports more dynamic risk assessment and precision-based care for ICU patients.

Our findings, combined with current international guidelines, highlight a potential role for GV in risk stratification (21). Future research is warranted to prospectively validate the observed thresholds and to explore the potential utility of integrating GV assessment into clinical management protocols.

We acknowledge several limitations of this investigation. First, the retrospective multicenter-cohort design carries inherent risks of selection bias and unmeasured confounding. Second, and most importantly, our analysis lacked adjustment for key stroke-specific prognostic factors (e.g., baseline NIHSS score, infarct topography, and details of reperfusion therapy) due to data unavailability in the databases used. Therefore, although GV remained significantly associated with mortality after extensive adjustment for available confounders, we cannot exclude the possibility that the observed association partially reflects residual confounding by the severity of the initial neurological injury. Relatedly, insulin therapy—a key modulator of glycemia—was not included in the primary multivariable model due to concerns about its potential role as a mediator on the causal pathway; consequently, its effect as a confounder was not fully isolated. It is important to emphasize that insulin use was markedly more frequent in the highest GV quartile (Table 1). This pattern suggests that elevated GV likely reflects a confluence of factors: greater underlying illness severity prompting insulin therapy, the treatment intensity itself, and potential iatrogenic contributions to glucose fluctuations. Therefore, while our model identifies an independent association, GV should be interpreted as a composite marker embedded within the clinical management context, and its precise causal contribution remains to be disentangled.

Third, the original and validation cohorts differed in their case definitions (ICD codes versus imaging-confirmed diagnosis), reflecting a pragmatic “broad-to-strict” validation strategy. While this affects direct comparability, it suggests the prognostic signal of GV is consistent across differing levels of diagnostic certainty. Fourth, the external validation cohort was small (*n* = 183), which compromises the precision of the effect estimates. The notably wide confidence intervals, especially for the highest risk quartile, reflect this uncertainty. While the consistent direction of association is valuable and aligns with the main findings, the hazard ratios in this cohort are unstable. Thus, the validation should be considered preliminary and hypothesis-generating. Fifth, our GV calculation relied on a minimum of three glucose measurements, a pragmatic choice that may influence estimate precision. The consistency of the association in analyses that adjusted for key sampling parameters (Supplementary Table S14) supports that the observed GV-mortality relationship is robust to such measurement variability.

Finally, GV should be interpreted as a composite marker, reflecting both intrinsic metabolic stress and external clinical treatments (e.g., vasopressors, mechanical ventilation). While adjusting for several such treatments strengthens the case for GV’s independent prognostic information, residual confounding from unmeasured factors (e.g., detailed insulin protocols) or from GV’s role as a marker of clinical instability cannot be excluded. Thus, while GV is a robust risk indicator, its precise causal role remains to be defined.

Despite these limitations, our findings robustly identify GV as a strong, time-dependent risk marker in critically ill ND-IS patients. Future prospective studies, incorporating stroke-specific severity indices and detailed treatment data, are needed to validate the observed thresholds, clarify the causal role of GV, and determine whether interventions to reduce GV can improve outcomes.

## 5 Conclusion

The original cohort using the MIMIC-IV database confirms that elevated GV is consistently and independently associated with an increased risk of all-cause mortality (ACM) in critically ill patients with non-diabetic ischemic stroke (ND-IS). This positive association was also observed in direction within a smaller, external validation cohort. Exploratory analyses across multiple time points suggested that the strength of the association between GV and mortality may increase over time. Furthermore, these analyses indicated a potential threshold effect, with the mortality risk exhibiting a nonlinear pattern that appeared to plateau at higher GV levels. The biological basis for this time-dependent and nonlinear association warrants further study. These findings add to the clinical evidence base regarding glucose management in critically ill stroke patients and highlight the value of further research to explore whether GV could be integrated into ICU metabolic risk assessment protocols.

## Data Availability

The raw data supporting the conclusions of this article will be made available by the authors, without undue reservation.
