# Supplementary material for: Prognostic significance and temporal patterns of glycemic variability in critically ill non-diabetic patients with ischemic stroke: a retrospective multicenter cohort study
Source: Front Neurol. 2026 Jun 12;17:1825235. doi: 10.3389/fneur.2026.1825235 (PMC13303125; doi:10.3389/fneur.2026.1825235)
Supplement: Supplementary file 1 [file Supplementary_File_1.DOCX]

**Supplementary Material OF Figures**

**Figure S1.** Cases with Missing Data Less Than 20%

**Figure S2.** Distribution of Glycemic Variability (GV) in the Study Population

**Figure S3.** Association of GV with ICU and Hospital Stays.​

**Figure S4.** Lasso Coefficient Trajectory Plot

**Figure S5.** Lasso Mean Squared Error (CVM) Trajectory Plot

**Figure S6.** Kaplan-Meier Curves at Different Follow-Up Time Points Stratified by Optimal Risk Stratification Cutoff Points in original cohort

**Figure S7.** Kaplan-Meier Curves at Different Follow-Up Time Points Stratified by Optimal Risk Stratification Cutoff Points in validation cohort

**Figure S8.** Spearman Correlation Matrix of Clinical Variables

**

**

**Figure S1.** Cases with Missing Data Less Than 20%

This figure illustrates the distribution and proportion of variables with missing data rates below 20% in the study cohort. Variables exceeding this threshold were excluded from analysis, and those retained were imputed using multiple imputation methods (m=5 datasets).

**
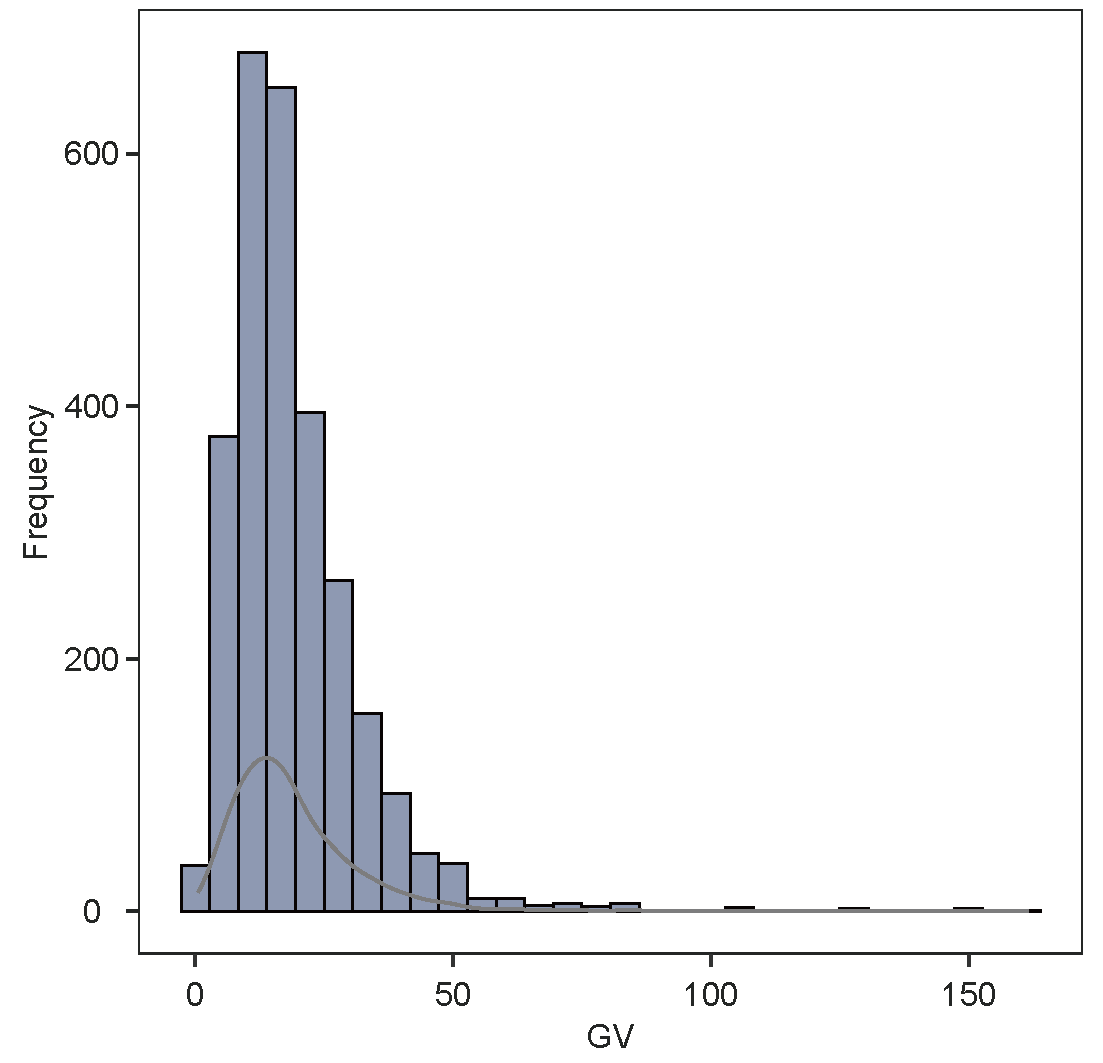
**

**Figure S2.** Distribution of Glycemic Variability (GV) in the Study Population

The histogram displays the frequency distribution of GV (quantified as coefficient of variation, CV) across the included non-diabetic ischemic stroke patients (n=2,788). The median GV was 16.27% (IQR: 10.80–24.10).


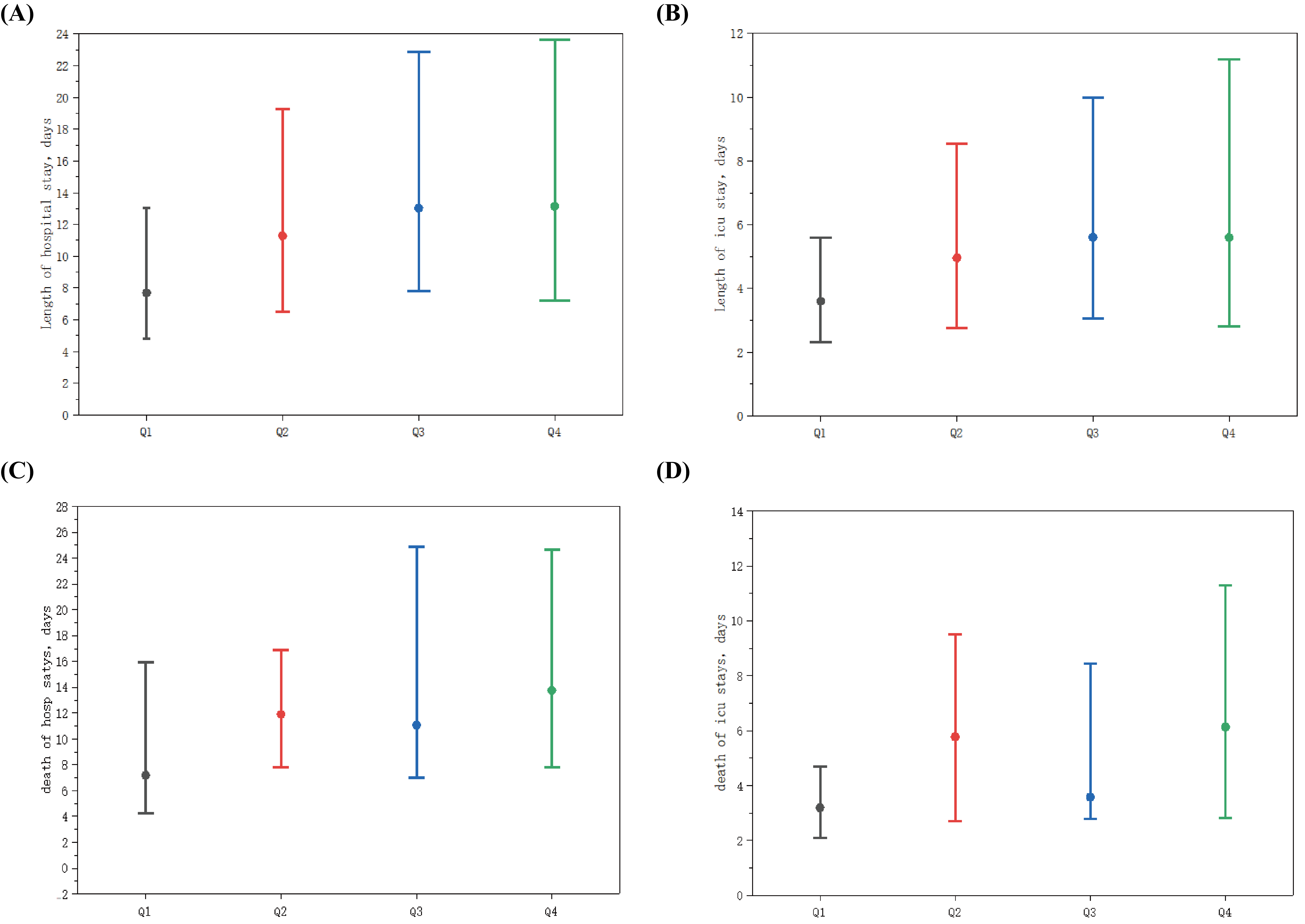


**Figure S3.** Association of GV with ICU and Hospital Stays. (A, B) Original cohort: (A) Hospital length of stay and (B) ICU length of stay across GV quartile. (C, D) Validation cohort: Corresponding (C) hospital and (D) ICU length of stay across GV quartiles.

**
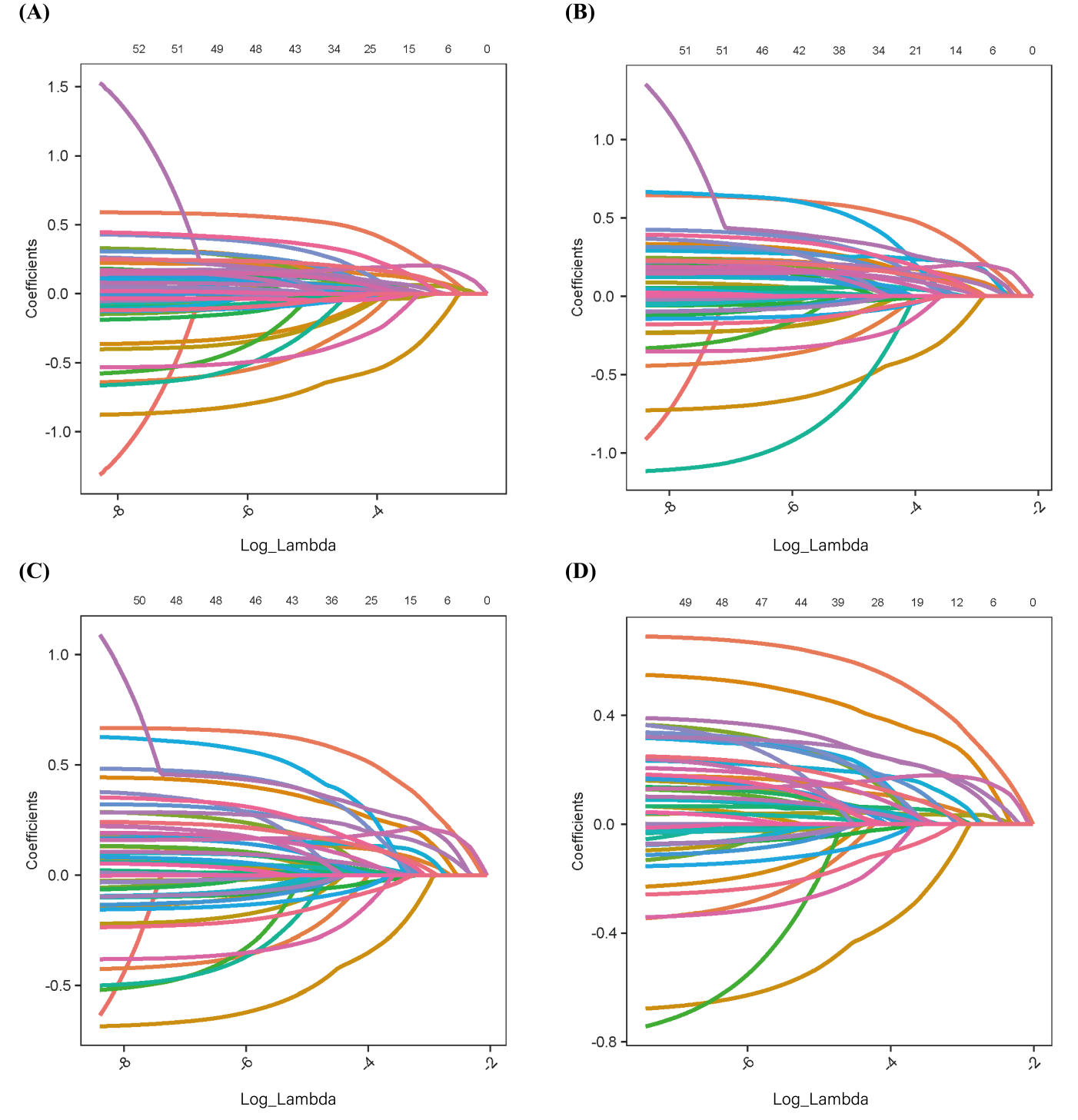
**

**Figure S4.** Lasso Coefficient Trajectory Plot

(A), (B), (C), and (D) correspond to the Lasso coefficient profile plots for the 28-day, 90-day, 180-day, and 365-day all-cause mortality prediction models, respectively. Combined with the mean squared error trajectory plot, it suggests that glucose variability (GV) is continuously retained in models with different follow-up times, and its impact on long-term mortality risk may become more significant over time.

**

**

**Figure S5.** Lasso Mean Squared Error (CVM) Trajectory Plot

(A), (B), (C), and (D) correspond to the Lasso mean squared error trajectories of the 28-day, 90-day, 180-day, and 365-day models, respectively. Combined with the coefficient plot, it suggests that glucose variability (GV) is continuously retained in models with different follow-up times, and its impact on long-term mortality risk may become more significant over time.

**

**

**Figure S6.** Kaplan-Meier Curves at Different Follow-Up Time Points Stratified by Optimal Risk Stratification Cutoff Points in original cohort

(A), (B), (C), and (D) correspond to the survival curves at 28 days, 90 days, 180 days, and 365 days of follow-up, respectively, showing that the all-cause mortality risk in the high GV group is significantly higher than that in the low GV group at all time points (all P < 0.001).

**Figure S7.** Kaplan-Meier Curves at Different Follow-Up Time Points Stratified by Optimal Risk Stratification Cutoff Points in validation cohort

(A), (B), (C), and (D) correspond to the survival curves at 28 days, 90 days, 180 days, and 365 days of follow-up, respectively, showing that the all-cause mortality risk in the high GV group is significantly higher than that in the low GV group at all time points (all P < 0.001).





**Figure S8.** Spearman Correlation Matrix of Clinical Variables

Heatmap of Spearman rank correlations among key clinical variables. High correlations (e.g., BUN vs. creatinine, ρ ≥ 0.95) led to exclusion of redundant variables to mitigate multicollinearity in subsequent modeling.
